# Formulation of zinc foliar sprays for wheat grain biofortification: a review of current applications and future perspectives

**DOI:** 10.3389/fpls.2023.1247600

**Published:** 2023-10-02

**Authors:** José Tonatiuh Sánchez-Palacios, David Henry, Beth Penrose, Richard Bell

**Affiliations:** ^1^ SoilsWest, Centre for Sustainable Farming Systems, Food Futures Institute, Murdoch University, Murdoch, Western Australia, Australia; ^2^ Chemistry, Murdoch University, Murdoch, Western Australia, Australia; ^3^ Tasmanian Institute of Agriculture, University of Tasmania, Hobart, Tasmania, Australia; ^4^ Research Institute for Northern Agriculture, Charles Darwin University, Casuarina, Brinkin, Northern Territory, Australia

**Keywords:** agronomic biofortification, wheat, foliar fertilizer, zinc, nanoparticles, silicon nanostructures, surfactants

## Abstract

Agronomic biofortification of wheat grain with zinc can improve the condition of about one billion people suffering from zinc (Zn) deficiency. However, with the challenge of cultivating high-yielding wheat varieties in Zn-deficient soils and the global need to produce higher-quality food that nourishes the growing population, innovation in the strategies to deliver Zn directly to plants will come into play. Consequently, existing foliar formulations will need further refinement to maintain the high agronomic productivity required in competitive global grain markets while meeting the dietary Zn intake levels recommended for humans. A new generation of foliar fertilisers that increase the amount of Zn assimilated in wheat plants and the translocation efficiency of Zn from leaves to grains can be a promising solution. Research on the efficacy of adjuvants and emerging nano-transporters relative to conventional Zn forms applied as foliar fertilisers to wheat has expanded rapidly in recent years. This review scopes the range of evidence available in the literature regarding the biofortification of bread wheat (*Triticum aestivum* L.) resulting from foliar applications of conventional Zn forms, Zn nanoparticles and novel Zn-foliar formulations. We examine the foliar application strategies and the attained final concentration of grain Zn. We propose a conceptual model for the response of grain Zn biofortification of wheat to foliar Zn application rates. This review discusses some physiological aspects of transportation of foliarly applied Zn that need further investigation. Finally, we explore the prospects of engineering foliar nano-formulations that could effectively overcome the physicochemical barrier to delivering Zn to wheat grains.

## 1 Introduction

Human zinc (Zn) deficiency is well documented. Since the recognition of Zn essentiality in humans during the second half of the 20th century (Hambidge, 2000), mild to moderate zinc deficiency has been noticed from the beginning of the 21st century in high-income populations resulting from suboptimal dietary Zn intake. Estimates indicate that suboptimal dietary intake of Zn affects 17% of the world’s population, including high-income countries in Europe and North America, and developing countries in regions of South Asia and Southern Africa (Wessells et al., 2012). In Australasia, where populations can access a relatively diverse diet, suboptimal dietary intake of Zn can affect primarily infants, adolescents, and the elderly (Gibson and Heath, 2011). Generally, the recommended dietary intake for Zn is around 15 mg per day, varying depending on lifestyle, age, gender, and diet types (Trumbo et al., 2001). Women during pregnancy and children are particularly susceptible to low Zn intake (Gibson, 2006). In developing countries, the prevalence of Zn deficiency is aggravated by frequent gut infections affecting the gastrointestinal absorption of Zn (Fischer Walker et al., 2013). Efforts to improve the food supply to developing countries have reduced the risk of Zn deficiency in 1.1 billion people from 22 to 16% (Kumssa et al., 2015). However, recent reports from the Food and Agriculture Organization of the United Nations indicate that restrictions imposed by the COVID-19 pandemic have increased the prevalence of Zn deficiency globally (FAO, I., UNICEF, WFP and WHO, 2020), because low-income people could not afford healthy diets to maintain optimal Zn levels (Herforth et al., 2020). Global inflation and rising food prices will lead to a decline in food security, which will impact a large portion of the population in poverty worldwide.

Zinc is an essential trace element for humans, playing a crucial role in catalytic, structural, and immune systems. It is particularly important for adaptive immunity (Bonaventura et al., 2015). Studies *in vitro* showed that Zn, in combination with cell membrane transporters, reduced the replication of various viruses, including influenza and SARS-coronavirus (Te Velthuis et al., 2010). Recent studies have shown that a human diet supplemented with Zn can increase the efficacy of current drugs tested clinically against COVID-19 (Derwand and Scholz, 2020). One effective and cost-efficient way to strengthen the immune system of the population against present and future viral infections, while also speeding up the recovery of immune-compromised individuals, is by consuming the optimal levels of vitamins and micronutrients including Zn.

### 1.1 Factors contributing to low dietary Zn uptake

One of the main reasons for low dietary Zn intake in humans is the cultivation of staple grains in soils with low plant-available Zn, resulting in Zn-deficient food. Zinc has been reported to be inadequate in 30% of agricultural soils globally (Alloway, 2008) and it is estimated that about 50% of soils used to cultivate grain are low in bioavailable Zn (Marschner, 1993). If applications are inadequate and availability is limited, it can lead to a decrease in Zn concentration in the edible portions of crops. Low bioavailability of Zn in agricultural soils is complex and can be associated with the lack of beneficial rhizosphere soil-microbe-root interactions (Rengel and Marshner, 2005; Rengel, 2015), sand texture, high pH, low levels of organic matter, low soil moisture, and high levels of chelating ions including phosphorus (Brennan et al., 2019). In Western and Southern Australia, extensive areas of arable land were Zn-deficient (Donald and Prescott, 1975). Historically, the application of Zn in these regions has been through the application of fertilizers containing trace amounts of Zn added in superphosphate and di-ammonium phosphate (DAP) at concentrations ranging from 400 to 600 mg of Zn/kg (Brennan, 2001). Historically Zn has been added to crops to achieve high-yielding potential but rarely the applied Zn accumulates in the filial tissue – the grain (Rengel, 2002). In the 1980s, the application of superphosphate fertilizer at 150 kg/ha containing Zn impurities was effective in maintaining adequate Zn levels in cereal crops, particularly when combined with high levels of N fertilizer after 15 years of application (Brennan, 1996). Due to this programme of supply, Zn deficiency in crop and pasture yields is now comparatively rare in southwestern Australia. Similarly, other countries such as Turkey, which previously had large areas of low Zn soils, have substantially corrected Zn deficiencies through farmer education programmes (Cakmak, 2008). Notwithstanding these impressive examples where Zn fertilizer increased yield, it is important to note that while soil Zn levels may be sufficient for achieving maximum yield, they may not be adequate for producing grain with Zn levels that meet the standards of human health (Cakmak and Kutman, 2018).

**Figure 1:**
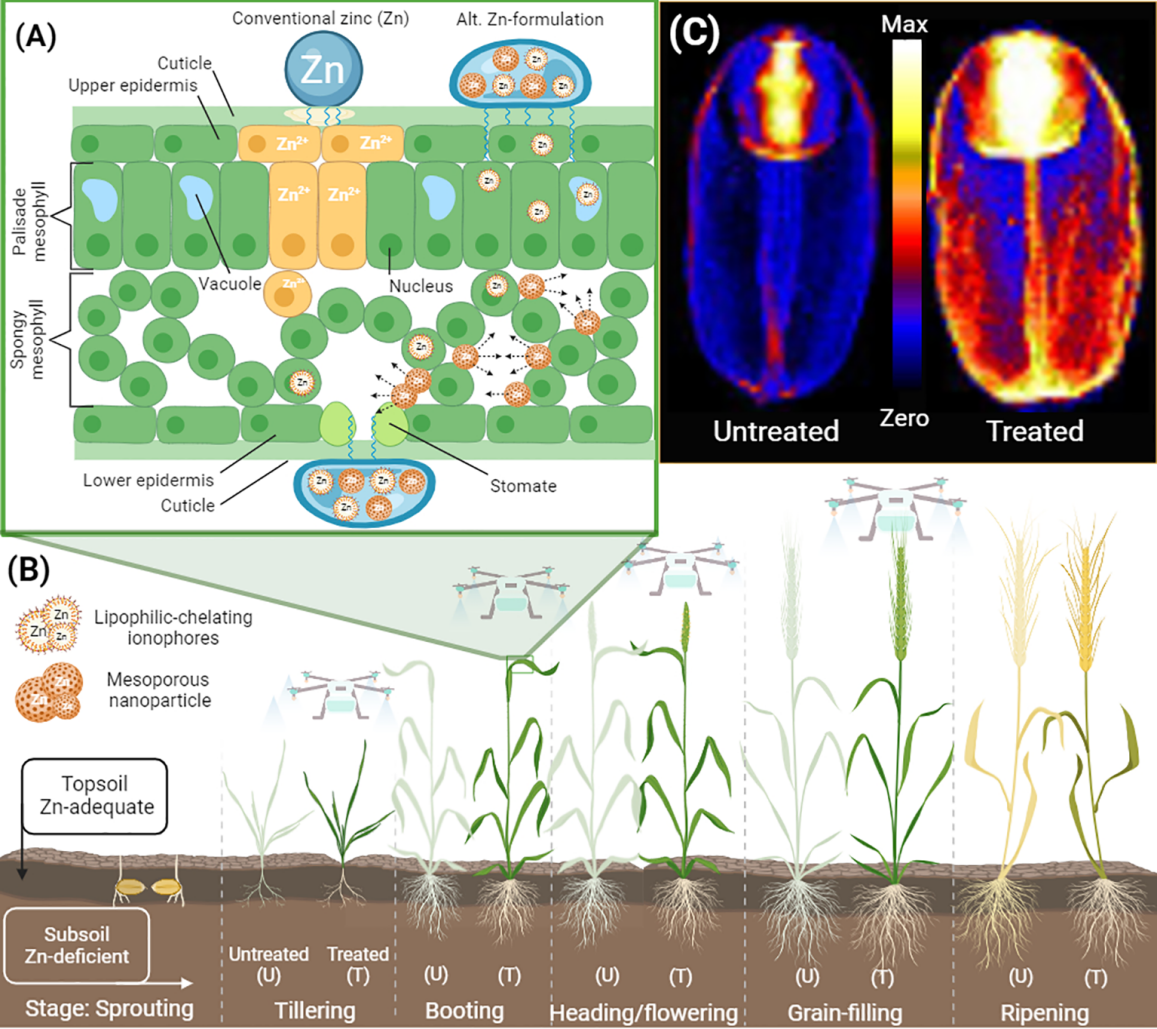
Leaf penetration model of alternative formulations with improved efficiency in delivering zinc (Zn) to wheat grains for agronomic biofortification. **(A)** A simplified drawing shows a cross-section of a flag leaf treated with conventional Zn and alternative Zn-formulation containing adjuvants, and mesoporous nanoparticles loaded with Zn and Zn lipophilic chelating agents known as ionophores. The advantage of alternative formulations over conventional is an increase in leaf penetration, reduction in Zn-toxicity and an increase in leaf mobility of Zn by the nature of the mesoporous nanoparticles and the ability of Zn-ionophores to penetrate cells and potentially move throughout the plant body unregulated. **(B)** Foliar application of Zn for the biofortification of grains can be performed from tillering to the grain-filling stage. Agronomic biofortification via foliar spray can be feasible in soils deficient in available Zn. Under such circumstances, the application of Zn foliarly during later stages can boost the concentration of Zn in grains. Drone technology allows for non-intrusive delivery of foliar formulations at later stages of development. **(C)** Alternative foliar formulations can result in wheat grain being biofortified with higher Zn concentrations in the endosperm compared to traditional formulations. The precise loading of Zn in various tissues of the wheat grain can be determined by X-ray Fluorescence Microscopy (XFM) techniques. The elemental maps of Zn in wheat grains (control and biofortified) were collected at the Australian Synchrotron. Graphics created with BioRender.com.

The restricted mobility of Zn in soil has significant effects on the Zn concentration in grains. In dryland environments, the stratification of Zn in soil can restrict root uptake. For example, surface application of Zn at relatively high rates, such as 22.5 kg Zn/ha, on acid sandy soil in southwest Western Australia remained in the topsoil layer even after 1430 mm of rain (Brennan, 1996). In southwest Western Australia, extractable Zn levels at 0-10 cm depth are typically adequate in agricultural soils, but due to the limited mobility of Zn, subsoil levels remain very low (Brennan and McGrath, 1988). Globally, little is reported about sub-soil Zn levels (Bell and Dell, 2008). Follett and Lindsay (1970) investigated a range of Soil Orders (Mollisol, Entisol, Alfisol and Aridisol) in Colorado, USA and found 35 out of 37 profiles had sub-soil Zn concentrations regarded as deficient. The implications of low sub-soil Zn were shown in a glasshouse study with two contrasting genotypes of wheat (*Triticum aestivum* L.), namely ‘Gatcher’ (Zn-inefficient) and ‘Excalibur’ (Zn-efficient), cultivated under inadequate subsoil-Zn (0.06 mg/kg DTPA-Zn). The study showed that grain Zn reflected inadequate subsoil levels in both genotypes (Nable and Webb, 1993). This indicates that even Zn-efficient cultivars may fail to extract Zn to enhance grain Zn concentrations from Zn-depleted soil. It is likely that young plants with shallow roots absorb Zn from the moist topsoil layer, but as the top layer dries out, Zn uptake by roots is more dependent on subsoil moist layers where available Zn remains very low (**Figure 1B**). A micronutrient survey conducted a decade ago on Australian crop soils confirmed that while 85% of soil contains moderate topsoil DTPA-Zn levels (>0.2 mg/kg DTPA-Zn) for crop yield, 55% of cultivated wheat grains in these soils had low grain Zn levels ranging from 10 to 15 mg/kg (Norton, 2013), an option not suitable for populations who follow a cereal-based diet.

### 1.2 Biofortification Of wheat with zinc

Biofortification of staple foods has emerged as a cost-effective approach to increase the density of desirable micronutrients in staple foods consumed by populations with limited access to diverse diets (White and Broadley, 2005; Nestel et al., 2006; Mayer et al., 2008; Palmgren et al., 2008; Norton, 2013). Biofortification can be achieved through conventional plant breeding, genetic engineering of crops or agronomic fertilization by soil or foliar application. Biofortification through plant breeding and transgenic approaches relied on sufficient genetic variation for a given trait. However, released varieties, especially for wheat, can be restricted in their suitability by variation among soil types and by the characteristics of a particular cropping system and, increasingly, by climate change (Cakmak and Kutman, 2018). Cultivars that accumulate high Zn in grains might also increase the risk of accumulating unwanted heavy metals in the grain when grown on contaminated land (Hussain et al., 2019), requiring careful consideration in biofortification programs. In order to ensure the safe development and implementation of interventions for biofortifying wheat grain with zinc, cost-effective cropping practices that complement existing methods may be necessary. The methods of farming have progressed, and now, the techniques of foliar application offer the potential alongside soil practices in a relatively short time frame to boost the nutritional content of grains grown in different soil types, especially in the face of changing climatic conditions.

Wheat is a major food crop cultivated on diverse soils and climates, yet it is a poor source of dietary Zn. Nevertheless, agronomic biofortification of wheat with Zn has been tested and proven to increase levels of Zn in grain. Evidence about applying Zn fertilizer to improve grain Zn concentration was first produced in Central Anatolia, Turkey (Yilmaz et al., 1997). In this study, Zn fertilizers applied to soil and foliage of wheat grown in field trials resulted in a 3.5-fold increase in the grain Zn concentration. Subsequent studies on Zn efficient varieties showed large genotypic variation among wheat lines with high Zn efficiency associated with enhanced capacity of soil Zn uptake, but the accumulation of Zn in leaf and grain per unit mass remained unchanged (Cakmak et al., 1999). Contrastingly, agronomic Zn foliar application studies have shown the potential for substantial and immediate improvement of grain Zn concentrations. For instance, Zn-foliar application studies found that the highest loading of Zn into grain occurred as a result of foliar application at the early milk stage of grain (10 days after anthesis), increasing the concentration of Zn from 45 mg/kg in the control to 55 mg/kg after 3 x Zn-foliar applications and to 70 mg/kg after 10 x Zn-foliar applications at a rate of 0.68 kg Zn per ha as ZnSO_4_ per foliar application (Ozturk et al., 2006). The first study testing the value of timing in foliar applications at booting and milk stages showed an increase of Zn from 28 mg/kg to 58 mg/kg when applied as ZnSO_4_ (Cakmak et al., 2010). Foliar application studies conducted in the North China Plain showed that when Zn was applied as ZnSO_4,_ an increase in whole wheat grain from 29 to 39.6 mg/kg Zn was observed, with an increase in white flour from 11.8 to 17.4 mg Zn/kg (Zhang et al., 2010). An international study testing Zn foliar application on wheat cultivars in 26 different field sites across Zambia, Thailand, China, India, Pakistan, Brazil, and Turkey, showed an increase in grain Zn concentrations from 28 mg/kg to 41.2 mg/kg on average (Ram et al., 2016). Most recently, a multi-year field study using high-yielding varieties of wheat from the Republic of Serbia showed that a single foliar application of ZnSO_4_·7H_2_0 at grain filling stage, increased grain Zn concentration from 15.9 to 20.5 mg/kg in the first season and from 26.4 to 32.6 mg/kg in the second season (Ivanović et al., 2021). A recent glasshouse study applying ZnO nanoparticles foliarly on wheat showed evidence of increased grain Zn content (Doolette et al., 2020). This study concluded that the application of conventional ZnSO_4_ to leaves can be as effective as applying ZnO nanoparticles. Combined application of Zn and iron (Fe) as a foliar spray (ZnSO_4_, 0.5% w/v; FeSO_4_, 1% w/v) showed an increase in grain Zn concentration to 55 mg Zn/kg, relative to the control with 40 mg Zn/kg (Ramzan et al., 2020). Indeed, a convincing number of Zn foliar studies in the past three years strongly indicates that foliar treatments can increase grain Zn concentrations. Nonetheless, the achievable target varies depending on Zn-soil availability, wheat cultivar and most importantly the rate and dose of Zn-foliar application employed (**Table 1**).

**Table 1:** Summary of recently published foliar studies for the biofortification of wheat grain with Zn.

Authors	Soil DTPA-Zn (mg/kg)	Wheat cultivar	Application rate, Stage(s) [Foliar formulation(s)]	Grain Zn control/increased (mg/kg)	Efficiency H/N/L/Null
(Ning et al., 2021)	0.91	Huaimai 22	2x, grain filling [ZnSO_4_ (0.4%, w/v), Zn-glycine and Zn+KH_2_PO_4_, Zn+insecticide, amino acid & fulvic acid, Tween 20 (0.01%, v/v)]	40/80	H
(Wang et al., 2023)	0.91	Huaimai 22	2x, flowering & grain filling [ZnSO_4_ (0.4%, w/v) Zn(Gly)_2_ (0.23%, w/v), pesticides, Tween 20 (0.01%, v/v)]	30/60-70	H
(Sattar et al., 2022)	n.a.	Bhakkar-2002	2x, tillering & flag leaf [ZnSO_4_ (5, 10, 15mM)]	27/40	H-N
(Ram et al., 2022)	1.2	HD2967 & PBW 725	2x, flag leaf & grain filling [ZnSO4 (0.5%, w/v), pesticides (thiamethoxam and propiconazole)]	25/35-50	H-N
(Dhaliwal et al., 2022)	0.62	16 genotypes	2x, tillering & flag leaf [ZnSO_4_ (0.5%, w,v)]	26-34/37-55	N
(Yu et al., 2021)	0.55-1.2	Winter wheat	2x, grain filling [ZnSO_4_ (0.4%, w/v) Tween (0.01%, v/v)]	22/40	N
(Sher et al., 2022)	n.a.	Zincol^*^, Fakher-e- Bhakkar & Faisalabed	3x, tillering,booting & heading [ZnSO_4_ (0.4% & 6%, w/v)]	16/25-36 mg/g content	N
(Sun et al., 2020)	0.6	LuoHan 6	4x, stem elongation, booting, flowering & grain filling [ZnO NPs (0.2%, w/v) & ZnSO_4_ (0.7%, w/v)]	18-23/39-40	N
(Zhang et al., 2017)	0.5	LuoHan 6	2x, stem elongation & early milk stages [ZnO NPs (0.2%, w/v) & ZnSO_4_ (0.7%, w/v)]	18-24/21-35	L
(González-Caballo et al., 2022)	0.2	Durum wheat	3x, tillering, stem elongation & flowering [ZnSO_4_ (0.9 mg Zn per plant), Tween 80 (0.5%, v/v)]	20-25/30-40	L
(Kiran et al., 2021)	n.a.	Faisalabad, Saher, Lasani & Punjab	4x, tillering, booting, early & late grain filling [ZnSO_4_ (0.2%, w/v)]	18-30/41-49	L
(Amaral & Brown, 2022)	0.25 µM ZnSO_4_	Patwin 515	2x, flag leaf [250 mg Zn/L & myo-inositol (0.25% v/v)]	55/60-70	L
(Jalal et al., 2022)	0.7	TBIO SOSSEGO	2x, tillering & grain filling [ZnO NPs, (0.75, 1.5, 3, & 6 kg Zn/ha)]	40-45/55-65	L
(Li-na et al., 2022)	0.98	Landraces & modern cultivars	3x, flowering, early & late grain filling [ZnSO_4_ (0.5%, w/v)]	44-47/50-59	L
(Ahmad et al., 2022)	0.4-0.6	Ujala-2016	2x, booting & grain filling [ZnSO_4_ (0.5%, w/v), boron and thiourea]	42/55-57	L
(Lian et al., 2023)	3.7	Zhengmai 10	3x, tillering, booting & grain filling [ZnSO_4_ (50, 100 & 250 mg/L), Zinc-glycerolate (Glyzinc)]	75/85-95	L
(Jalal et al., 2023)	0.7	TBIO SOSSEGO	2x, tillering & grain filling [ZnO NPs 150nm (0.75, 1.5, 3, & 6 kg Zn/ha) seed inoculation]	33/40	L
(Naz et al., 2023)	1.09	Galaxy-2013 & Faisalabad-2008	3x, tillering, booting & flowering ZnSO_4_ (0.5% & 1%, w/v)	59-65/70-77	L
(Ahmad et al., 2023)	0.81	Wadan-2017	Rate and stage – n.a. [ZnSO_4_ (0.2%, w/v), ZnO nanofiber (0.016%, w/v; 25 nm)]	21/33	L
(Ivanović et al., 2021)	0.88-1.28	Talas, Ratarica, NS 40S, Dika & Simonida	1x, flowering [ZnSO_4_ (0.5%, w/v)]	21/26	L
(Zhang et al., 2021)	1.1	Yumai	4x, jointing, booting, flowering & grain filling [ZnSO_4_ (0.3%, w/v)]	21/21	Null

n.a., information not available; *improved wheat variety for Zn-biofortification.

H, high efficiency; N, normal efficiency; L, low efficiency & Null, null efficiency. The foliar formulation gives details of the form of Zn and additives. Grain Zn concentration is given in mg/kg unless stated otherwise. Studies are ranked based on the model of foliar efficiency proposed herein.

In Australian wheat, little is known about the use of Zn-foliar application practices for the biofortification of wheat grain. A study in southwestern Australia tested the effectiveness of Zn foliar application in preventing Zn deficiency in wheat before the tillering stage and found that the form of Zn-EDTA was more effective compared to ZnSO_4_ for increasing plant growth, but there were no differences between Zn forms at later stages of growth: the effect of Zn-foliar on wheat grain was not reported (Brennan, 1991). Other studies in South Australia have tested the role of Zn-foliar application to reduce the accumulation of concerning contaminant metals such as cadmium (Cd) in grain and suggested that high rates of Zn-foliar application were needed to reduce the accumulation of Cd in wheat grain (Oliver et al., 1997).

Given the prevalence of low Zn intake in human diets and the effectiveness of Zn-foliar application for biofortification of wheat grain, uncertainty about the best methods and Zn forms for foliar application presents an opportunity to develop agronomical practices for low Zn soils, especially those with inherently low sub-soil levels of Zn, to produce Zn biofortified wheat grain with nutritional benefits for the human diet. As the subject of foliar Zn bio-fortification has been reviewed in numerous articles recently (Bhatt et al., 2020; Rehman et al., 2020; Singh et al., 2023), the following review will focus on opportunities to develop novel materials and delivery systems for biofortification of wheat grain. In the following sections, we will be discussing the effective delivery of both Zn-nanoparticles and emerging nanostructure carriers for uptake by leaves and loading of Zn to grain, with a specific focus on wheat.

### 1.3 Nano-fertilizers and nanomaterial for micronutrient delivery

More efficient approaches for the foliar application of micronutrients to crops on the broadacre scale can be achieved through the production of novel fertilizers and delivery processes. Crop production can benefit from nanoparticle properties due to their initial fast reactivity, sustained slow-release, and surface-coating effects for intervascular transfer to developing tissue (Gutiérrez et al., 2011). Moreover, the use of natural materials to produce nanomaterials that can be used as vehicles for loading and delivering micronutrients to grain could reduce the risk of toxicity in plants. The introduction of nanotechnology in agriculture can alleviate the problems associated with suboptimal Zn intake in humans, which without dietary diversification will remain a challenge. Nano-fertilizers are defined as nutrients or nutrient-containing materials in the nanometer scale (< 100 nm) that can be used for the delivery of micronutrients to crops in a controlled and optimized manner (DeRosa et al., 2010). The following section reviews novel materials and delivery processes that could improve the efficacy of Zn foliar application on crops for increasing the concentration of grain Zn.

## 2 Zinc oxide nanoparticles (ZnO-NPs)

Over the last few decades, nanoparticles of Zn have attracted global attention and have progressively been used as soil and foliar nano fertilizers. Zn oxide (ZnO) is a white, water-insoluble powder. ZnO nanoparticles (ZnO-NPs) can be synthesized by a range of methods including spray pyrolysis, thermal decomposition, chemical vapor deposition, laser ablation and green synthesis using plant extracts such as rice flour (Ramimoghadam et al., 2013; Sabir et al., 2014). A study using lysimeters and wheat plants has shown that the application of ZnO-NPs to soil can be effective at increasing Zn levels in grain (Du et al., 2011). In this study, 110 kg of loamy clay soil spiked with 5 g of ZnO-NPs increased the concentration of Zn in wheat grain to 60 mg Zn/kg compared to the control with 40 mg Zn/kg. However, above-ground biomass was reduced by 7.6%, most likely as a result of high levels of available Zn from excessively high rates of ZnO-NPs application. A different glasshouse experiment showed that 1000 mg of ZnO-NPs/kg in soil increased the concentration in grain from 18.3 mg Zn/kg in control to 60.4 mg Zn/kg in ZnO-NPs treated plants, while application of moderate levels of foliar ZnO-NPs (20-100 mg/kg) increased aboveground biomass by 62% and grain yield by 55% (Du et al., 2019). A field trial using the wheat variety Luo Han 6 in the Shaanxi province of China showed that foliar application of ZnO-NPs (>100 nm in size) at 0.96 kg Zn/ha caused a small but significant increase of Zn concentration in grain relative to the control. During two consecutive cropping seasons, Zn concentration increased from 18.4 to 26.5 mg Zn/kg in 2016 and from 23.6 to 34.6 mg Zn/kg in 2017 (Zhang et al., 2017). Synchrotron studies of Zn-biofortified wheat grain from the second cropping season revealed that foliar application of ZnO-NPs had no effect on the distribution and speciation of Zn in the endosperm, nonetheless, additional Zn was identified as Zn phosphate, which can be assimilated in humans. A subsequent field trial using the same wheat var. Luo Han 6 at the same research station applied smaller ZnONPs to leaves, resulting in a 1.65-fold increase in grain Zn concentration of 38.6 mg Zn/kg in the first 2017 season, and a 2.2-fold increase in grain Zn of 40.2 mg/kg in the second 2018 season (Sun et al., 2020). In this study, synchrotron-based X-ray fluorescent microscopy (XFM) analysis revealed a 20-fold increase of Zn in grain endosperm from 1.5 mg Zn/kg in control to 30 mg Zn/kg. Recent efforts applying ZnO-NPs have shown a moderate increase in grain Zn concentrations relative to the control attaining a maximum of 65 mg Zn/kg after 1.5 kg of Zn foliar per ha application under wet tropical conditions (Jalal et al., 2022). Overall, these studies suggest that Zn applied as ZnO-NPs can be employed as a fertilizer to increase the Zn concentration in wheat grain. However, the efficacy of ZnO-NPs depends on application rates, nanoparticle size and most importantly confirming the advantages of ZnO-NPs over conventional forms of Zn such as ZnSO_4_. More comparative studies between ZnO-NPs and conventional Zn forms as foliar fertilizers are needed to establish the real benefits and improvements of using Zn nanoparticles as a tool for wheat grain biofortification.

## 3 Nanomaterial-enhanced fertilizers

An additional approach involves the application of nanostructures enriched with fertilizers to enhance crop productivity and biofortify grain. Nanomaterial-enhanced fertilizers are defined as nanomaterials with internal nanostructures used for nutrient loading, and when applied to soil can function as slow-release fertilizers (Liu and Lal, 2015). Enhancing nutrient efficiency in soil by applying nanostructures is a method that has been tested for many decades with the application of zeolite to the soil. Zeolites are a class of natural, porous, aluminosilicate compounds with a range of compositions, which have well defined structures with regular nanopores and interconnected voids (Flanigen, 1980). The application of zeolite to agricultural soils has shown benefits in improving soil water and nutrient retention including nitrogen (N) (Nus and Brauen, 1991; de Campos Bernardi et al., 2016). Although the application of naturally occurring nano-minerals such as zeolite has benefitted agriculture in challenging soils, recent advances in nanocarriers used as drug delivery vehicles in human therapy opens the door to achieving significant efficacy in delivering micronutrients to edible parts of crops (Avellan et al., 2019; Lowry et al., 2019; Sanzari et al., 2019). The following section discusses the application of commonly used porous nanoscale materials, namely hydroxylapatite, silica, and chitosan as vehicles to deliver fertilizers to crops.

### 3.1 Hydroxylapatite nanoparticles

Hydroxylapatite (or hydroxyapatite, HAp) is the inorganic component of the hard tissue of vertebrates represented by the formula Ca_10_(PO_4_)_6_(OH)_2_. HAp nanoparticles (HAp-NPs) can be synthesized by thermal dry or wet methods. Dry methods require a relatively high temperature above 700°C followed by grinding or milling to obtain the required size of nanoparticles. Anti-sintering agents can be used to prevent melting (Okada and Furuzono, 2012). Wet methods require precipitation in solution and imposed conditions such as ionic strength, pH and temperature that dictate the shape and size of HAp-NPs. Morphology and precipitation of HAp-NPs also depend on the presence of surfactants in the solution (Okada and Furuzono, 2006).

The application of HAp-NPs in crop science was first tested as a source of P fertilizer in soil media using soybean plants (*Glycine max*) in a pot study (Liu and Lal, 2014). In this study, wet-synthesized spherical HAp-NPs with a diameter of 15.8 ± 7.4 nm increased growth rate and seed yield by 32.6% and 20.4%, respectively, in comparison with conventional P fertilizer applied as Ca(H_2_PO_4_)_2_. Interestingly, below-ground biomass was enhanced by 41.2% in the presence of HAp-NPs. The first study to incorporate micronutrients such as iron (Fe), copper (Cu), boron (B) and Zn (II) oxide (ZnO) into HAp-NPs and test them as a soil fertilizer was carried out using *Asparagus officinalis* (Phan et al., 2019). In this study, HAp-NPs were prepared by a chemical reduction process in solution. Micronutrients were then integrated into the surface by coating and short-chain alginate was used to obtain the final nanostructure resulting in rod-shaped nanoparticles of 100-150 nm length and 20-25 nm diameter. Application of HAp-NPs with micronutrients to seeds resulted in accelerated germination after 10 days and seedlings irrigated with nanoparticles grew faster compared to the control. An independent study incorporating metal nanoparticles of Cu, Fe and Zn to urea-modified HAp-NPs showed an increase in the concentration of micronutrients in fruits of treated ladies’ finger (*Abelmoschus esculentus*) plants (Tarafder et al., 2020). In this study, nanostructures 38.21 nm in size were applied to soil media at a rate of 50 mg of HAp-NPs in 250 mL of tap water in two different events for 14 days. A similar approach was carried out in corn seeds (*Zea mays*), where the application of Zn-loaded urea-HAp-NPs increased germination and growth rate by 19% and 69%, respectively, compared to control (Abeywardana et al., 2021). Currently, there is a lack of studies exploring the potential benefits of using HAp-NPs to biofortify wheat grains with Zn.

### 3.2 Silica nanoparticles

Silicon oxide or silica (SiO_2_) is naturally found in the environment as quartz and in living organisms including plants. While SiO_2_ is not essential for all higher plants, increasing evidence shows that Si uptake can alleviate toxic effects associated with abiotic stress from salinity, drought, and heavy metals and increase resistance to fungal infection (Luyckx et al., 2017). Silica uptake in wheat is suppressed by metabolic inhibitors, dinitrophenol and potassium cyanide, suggesting active regulation by plants (Rains et al., 2006). Foliar application of bulk silicate (40 g/L) on rice showed a decrease in infection intensity by *Bipolaris oryzae*, a fungus that causes brown spots in rice; however, soil application was more effective compared to a foliar application (Rezende et al., 2009). A comparative study on the effect of foliar application of non-porous silica nanoparticles (20-40 nm) versus bulk silica on phytochemical responses in maize showed that foliar silica nanoparticles enhanced the expression of essential phenolics, however, again soil application was more effective than foliar applications (Suriyaprabha et al., 2014).

In contrast, mesoporous silica nanoparticles (MSNs) have been considered good candidates for use as nanostructure carrier systems because of their unique properties namely, hydrophilicity, pore size, surface area, surface functionalization, physical and chemical stability including biocompatibility and degradability under physiological conditions (Hussain et al., 2013; Hartono et al., 2014; Yi et al., 2015). Currently, MSNs have been used largely as biomolecule delivery vehicles in human cells, in particular in drug delivery agents for cancer treatment (Mohamed Isa et al., 2021). Hence, MSNs have great potential to be employed as carriers of nutrients in plant cells. The first study to demonstrate the uptake and accumulation of MSNs in various plants including lupin, wheat, maize and the model plant, Arabidopsis, showed clear evidence that MSNs were able to penetrate cell walls and membranes to reach vascular tissue to be transported to aerial parts of plants (Sun et al., 2014). In this study, monodisperse silica nanostructures, 20 nm in size with interconnected pores of 2.5 nm, were visualized using a combination of confocal laser scanning microscopy and proton-induced X-ray emission (micro-PIXE). A fluorescent marker for tracking MSNs in tissue revealed uptake and distribution patterns in these plants. A subsequent study showed that wheat and lupin plants exposed to levels of MSNs up to 200 mg/L were unaffected, which demonstrates the capacity to apply MSNs in high concentrations (Sun et al., 2016). Mitotic studies of root tip cells of *Lens culinaris* exposed to a range of concentrations of commercially available MSNs (AEROSIL 300) reported that lower concentrations ranging from 25 to 50 mg of silica/L promoted plant growth. However, higher concentrations ranging from 200 to 300 mg of silica/L led to abnormalities in mitosis (Khan and Ansari, 2018). To date, MSNs have not yet been used as nutrient carriers but rather applied as a source of Si in crops. With the development and application of MSNs in other disciplines, it is anticipated that MSN technology will have beneficial outcomes in agronomic systems including the potential for improving Zn delivery to plants and grains.

### 3.3 Chitosan nanoparticles

The application of chitosan nanoparticles (CNPs) in agriculture is motivated by the need for cost-effective and sustainable production of agrochemicals (Kashyap et al., 2015). Polysaccharide-based nanoparticles such as CNPs are known to be environmentally friendly, non-toxic and biodegradable (Divya and Jisha, 2018). Chitosan is derived from chitin, which is the second most abundant polysaccharide after cellulose. Chitin is the major component of the cell walls of fungi and the exoskeleton of arthropods and fish scales (Yanat and Schroën, 2021). There are several different methods to synthesize CNPs. The first method involves the use of the glutaraldehyde crosslinking technique for the preparation of chitosan nanoparticles by forming covalent attachments (Ohya et al., 1994). Alternatively, the microemulsion approach uses water-in-oil reverse micelle structures (Kafshgari et al., 2012). Additional methods for the synthesis of CNPs include precipitation-based (Tokumitsu et al., 1999), ionic gelation (Calvo et al., 1997), radical polymerization (Hu et al., 2002), and top-down methods (Wijesena et al., 2015). The ionic gelation technique has also been employed for the synthesis of CNPs and encapsulation of pesticides (Grillo et al., 2014). In this approach, where CNPs of 300 nm encapsulated the herbicide paraquat, its effectiveness increased while the impact on the environment was reduced. Alternatively, CNPs (500 – 700 nm in size) loaded with nitrogen, phosphorous, and potassium (NPK) were used for foliar application on wheat (cv. Egypt-1) at 500 mg N/L, 60 mg P/L and 400 mg K/L in both normal and CNP forms (Aziz et al., 2016). This study showed that NPK-CNPs were taken up by leaves and that nanostructures were observed in phloem sieve tubes. The application of NPK-CNPs shortened the life cycle of wheat from 170 to 130 days. A study on grain quality with the same treatments showed a reduction of grain N levels when treated with high strength of NPK-CNPs relative to control (Abdel-Aziz et al., 2018). Most likely, the reduction in the life cycle affected the uploading of N in grain. A pot study using sand irrigated with Zn-free Hoagland solution, tested the application of Zn-loaded CNPs for foliar application to durum wheat cultivars after anthesis and showed the concentrations of grain Zn in both cultivars (~ 30mg Zn/kg) increased relative to control (20 mg Zn/kg) (Deshpande et al., 2017). In this study, the localization of Zn-CNPs in leaf tissue was determined in vascular bundles using fluorescence microscopy and Zinquin dye. However, localization of Zn in grain using the diphenyl thiocarbazone (DTZ) method gave ambiguous results. This study did not include a foliar treatment with a conventional form of Zn (e.g., ZnSO_4_), so the relative effectiveness of the foliar Zn-CNPs treatment cannot be determined. A subsequent field study by the same authors showed that foliar application of Zn-CNPs to wheat plants increased the concentration of Zn in grain to 53.3 mg/kg when applied at 40 mg Zn/L relative to control with 39.5 mg Zn/kg in grain (Dapkekar et al., 2018). In comparison, Zn application as ZnSO_4_ at a ten-fold higher rate (400 mg/L), produced grain at 59.4 mg Zn/kg. Since the amount of Zn loaded in CNPs was 10 times lower, the Zn-nanostructure delivery technology was more efficient and cost-effective. It should be mentioned that all Zn treatments contained N as urea which may have confounded the results since grain from the N-only treatment also showed an increase in Zn levels relative to control (42.8 mg Zn/kg and 39.5 mg Zn/kg, respectively).

### 3.4 Using adjuvants to increase Zn uptake from nanoparticles

Agricultural adjuvants, chemicals that aid the uptake of active ingredients in chemical formulations, have been used extensively to increase the efficacy of pesticides, microbial inoculants, and fertilizers (Kovalchuk and Simmons, 2021). Leaf physical and chemical properties such as trichome density, stomatal density and stomatal pore size and the presence and nature of epicuticular waxes can affect the uptake of foliar-applied Zn (Xie et al., 2020), and therefore adjuvants such as surfactants and oils can be used to increase leaf penetration (and therefore plant uptake) of foliar-applied Zn.

The use of adjuvants to increase foliar uptake of Zn has been investigated for several decades. Crowley et al. (1996) investigated combining the application of three forms of Zn (ZnSO_4_, ZnO and Zn metalosate) with three commercial surfactants (Kinetic, Sun-It II and Tween 20) and a water treatment to the leaves of avocado trees (*Persea americana* Mill.). They found that there was no difference in Zn uptake for all the Zn-adjuvant combinations except for Sun-It II and ZnO, where combining the Zn source with the adjuvant more than doubled Zn leaf concentrations. Haslett et al. (2001) applied four forms of Zn to young wheat plants (ZnSO_4_, ZnO, ZnEDTA and Biomin Zn) with a surfactant (Agrol-600) and found that adding the surfactant significantly reduced plant growth. In a separate experiment, they combined Agrol-600 with radio-labelled elemental Zn^65^ and found the surfactant increased leaf uptake of Zn by 15%. It is clear from the literature that the efficacy of adjuvants to increase Zn uptake is specific to the combination of the form of Zn used as well as the adjuvant type. To our knowledge, there have not been any studies where adjuvants have been used in conjunction with Zn nano-fertilizers to increase foliar uptake, and therefore we recommend this as an area of future study.

Foliar formulations containing additional components such as adjuvants and other bio-stimulants can indeed have a positive effect by increasing the Zn leaf content consequently increasing the potential of translocation of zinc from leaf to grain. Further research needs to be conducted to fine-tune the use and application of adjuvants in foliar formulations. Studies should aim for the following: i) to identify the mechanism of leaf penetration using selected adjuvant products and different zinc forms to establish compatibility, ii) to determine the threshold concentration of adjuvants causing leaf cuticle damage leading to propensity to air-born infections resulting in yield losses, iii) heterogeneity of formulations can result in significant variation in spraying Zn concentrations, therefore miscibility of the formulation should be investigated. The latter is important as variation in grain Zn concentration can be attributed to Zn forms, wheat genotype and environmental constraints, apart from variation in the formulation *per se*.

Furthermore, more sustainable products are commercially available. Efforts should be made to use biodegradable products for foliar spray studies. Traditional petrol-based adjuvants such as Tween have been used widely for their benefits in improving leaf surface contact are (Ning et al., 2021; Yu et al., 2021; González-Caballo et al., 2022; Wang et al., 2023). However, more sustainable, and greener surfactants should be considered for further testing in biofortification trials.

## 4 Sustained controlled release formulation

The mechanism(s) responsible for the transport of foliarly applied Zn to grains can be attributed to the mechanisms described in the remobilization of Zn recently reviewed by Xia et al. (2020).In this review, evidence was discussed for the transcription factor (*NAM-B1*) playing a key role in accelerating senescence in leaves, which resulted in an increase in the translocation ratios of Zn from leaves to grain (Uauy et al., 2006). Uauy et al. (2006) used RNA interference molecular technology to downregulate the RNA levels of the NAM genes family and detected a 30% decrease in grain Zn concentration relative to the null treatment. A study in flag leaf revealed that GPC1 contributes to upregulating genes associated with the active loading of Zn into the phloem namely zinc transporter protein (ZIP) and yellow stripe-like (YSL) family of proteins (Pearce et al., 2014). A previous review concluded that the main barrier for Zn reaching the grain is the transfer from the xylem to the phloem at the base of each grain (jan Stomph et al., 2009). Stable isotope tracing studies confirmed that the primarily bottleneck barrier for Zn transport into wheat grains is the connecting tissue between stem rachis and the grain (Wang et al., 2011). This suggests that wheat grain Zn accumulation is restricted by the mechanisms of transport to the grain vascular tissue, which requires alternative means of delivery.

Careful design of nanostructures that function as nutrient carriers will allow for the controlled release of Zn so that this is matched with the active transporting mechanisms in leaves resulting in an increase in the transfer of xylem to phloem near the grain. Carbon-based slow-release nanomaterials are gaining significant attention as delivery vehicles of fertilizers due to their small size and increased efficacy in delivering nutrients while reducing fertilizer loss such as N and Zn in wheat plants. Alikhani et al. (2023) applied Zn and carbon-dot nanocarriers to soil in pots over 30 days under controlled conditions resulting in a 19% Zn increase and 118% N increase in grain compared to conventional ZnSO_4_. This study adds to pioneering studies demonstrating that the porosity and surface area of nanostructures can be manipulated by physical or chemical methods, which allows for nutrient loading and controlled release. In that way, targeted and more efficient nutrient release becomes possible, allowing for Zn biofortification in grains together with a reduction of application rate and costs.

Foliar application has distinct advantages over soil application because it ensures direct contact of the Zn-loaded nanostructures with the plant shoot. Once on the leaves, absorption of the nanoparticles and/or their Zn payload can occur through the stomata and the epidermis (**Figure 1**). The absorption of nanoparticles via these different pathways will be influenced by the size, shape, and surface chemistry of the particles. Incorporation of specific functionality that enhances either the lipophilicity or hydrophilicity on the surface of the nanoparticle can preferentially increase adsorption via one or other of the pathways. However, biofortification also requires the transport of nanoparticles and/or their Zn-payload to the grain. Once absorbed, nanoparticles are then able to diffuse through the extracellular spaces (apoplast) or across the cell membrane (symplast). The size of the nanoparticles has an impact on the transport pathway. The apoplast is large enough to facilitate the diffusion of particles in the 50 – 200 nm size range. Smaller particles can more easily pass through cell membranes and are therefore transported by the symplast (Hong et al., 2021). Endocytosis is one mechanism by which NPs can enter cells, but appropriate functionalization of NPs may facilitate transfer across cell walls by other mechanisms. For the long-distance transport in wheat, NPs could be transported via the xylem or the phloem (Notaguchi and Okamoto, 2015). The xylem and phloem transport various materials (water, nutrients, sugars) throughout the plant. This may result in the NPs being transported to the roots rather than the grain or increased accumulation in stems (nodes and internodes) and old leaves during vegetative growth or maximize grain load capacity if NPs are applied at later stages of plant development. Controlled release for biofortification requires the loaded NPs to be able to navigate these various pathways to the grain and then release their micronutrients into the grain or closest to the separation space between the maternal and the filial tissue. The rate at which the Zn is released from the nanoparticles is an important aspect of sustained release as the grain develops. A slow-release rate could be beneficial in gradually increasing the Zn delivery via the crease phloem to the developing grain while avoiding reaching levels that would be toxic to the plant. In the contrary, if the release rate is too low this may lead to inefficiency of biofortification of grains. Therefore, the application of nanostructures with slow-release of Zn for biofortification require further study.

## 5 Framework for comparing foliar biofortification methods

**Figure 2:**
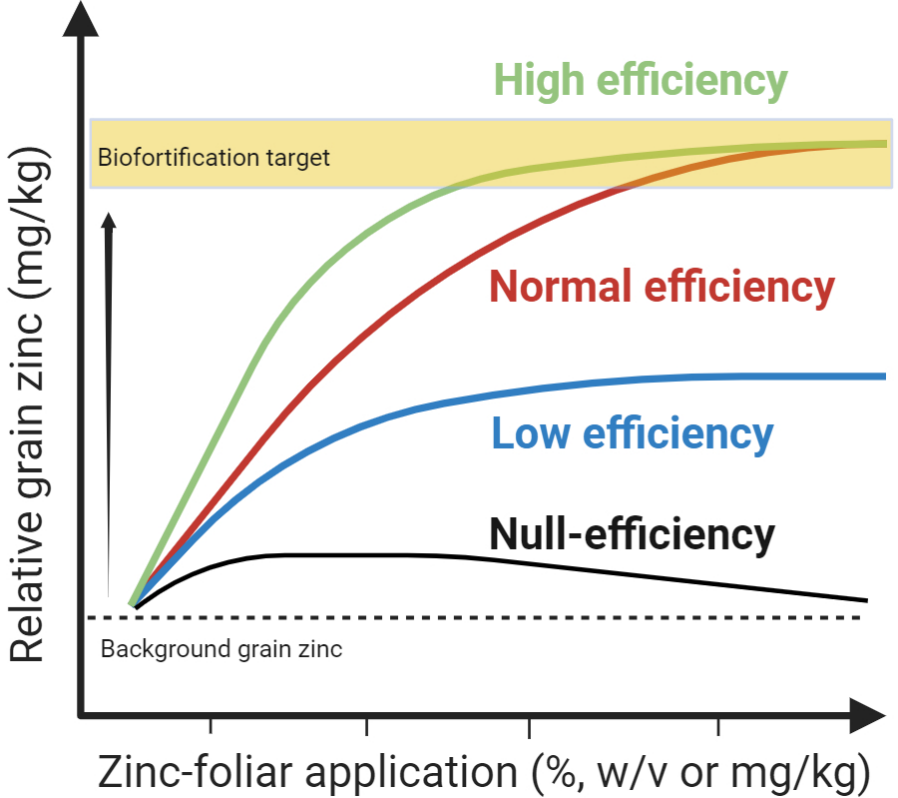
Conceptual curve model describing the increase in zinc (Zn) accumulation in wheat grain derived from Zn foliar application. Based on this model a classification of the use efficiency of foliar formulations is proposed. The high-efficiency formulation curve has increased grain zinc amplitude, reaching the desirable target with a low Zn-foliar application with a relatively low grain Zn concentration. In contrast, a normal efficiency formulation curve follows a reduced amplitude pattern and a slower rate of grain Zn accumulation from foliar events requiring a higher foliar dose to reach the target. Low-efficiency formulation curve has low amplitude and several Zn-foliar application events of a higher concentration would be required– the target may not be attainable. Null efficiency curve has minimal to no amplitude regardless of the concentration and frequency of zinc-foliar applications and in some instances, it may be toxic. Note the degree of efficiency may vary depending on the background of grain Zn concentration before foliar application. Graphics created with BioRender.com.

To optimize the Zn foliar methods, efforts should be directed towards determining the efficiency of selected foliar formulation for a given wheat variety cultivated in a specific or across multiple agroecological regions. Based on recent studies (**Table 1** and above), a quadratic model can be applied to describe the relationship between total foliar dose delivered in a single foliar application or in a sequential series of zinc-foliar dose fractions and linking this with the biofortification of wheat grain (**Figure 2**), with the assumption of interaction between foliar dose and relative grain Zn concentration. The quadratic model allows for a comparison of the effectiveness of different foliar formulations and protocols used under different environmental constraints such as soil and variety type. In this model, wheat developmental stages and soil types are factors of interest or covariable that influence the likelihood of increasing or decreasing the Zn levels in grain from foliar sprays as observed in the study by González-Caballo et al. (2022). The curve model also allows for the comparison of formulations delivered in variable dose fractions. Each fraction can be added to calculate the overall total dose. It is possible that some formulations may increase their Zn-foliar use efficiency if the total dose is fractionated into variable dose applications. Ideally, the optimal method to achieve the desirable grain Zn concentration target for biofortification should result from applying the minimum amount of total Zn-foliar dose preferentially in single or double foliar fractionation during key developmental stage(s) assisted by potent additives or biostimulants in the formulation to increase leaf penetration and Zn delivery to grains (**Figure 1**). Fine-tuning of agronomic biofortification methods would translate into a low operational cost making this approach more attractive for farmers growing grain products for markets seeking Zn-enriched grains.

## 6 Future perspectives and conclusion

Foliar application of nano-fertilizers for the biofortification of grains with nutrients is a promising but still inadequately tested approach to improve the health and well-being of millions of people. Animals that eat grains can also benefit from consuming biofortified grains. The primary benefit of foliar application in agrosystems is the delivery of micronutrients directly to plants resulting in an optimized use-efficiency delivery method of fertilizers for crop production. Compared to conventional methods, foliar application demonstrated equal or better efficiency of Zn delivery to crops. In the face of changing soil management practices (e.g., no-tillage), foliar application of Zn fertilizers can be a viable strategy for delivering nutrients to above-ground parts when needed without reliance on soil disturbance. Furthermore, foliar delivery of nutrients can be applied systematically to boost the concentration of nutrients in grains to alleviate not only persistent deficiencies of micronutrients - endemic in many developing countries - but also ameliorate the emerging issue of suboptimal nutritional intake globally (i.e., in both developed and developing countries). Even though foliar applications have been widely studied and applied in horticulture, additional research is needed to create new formulations that can optimize nutrient use efficiency in crops and loading in grain. Nutrient use efficiency is critical in the evaluation of crop production systems, which determines how well crops use the available nutrients. Foliar application of nutrients can enhance nutrient acquisition, biomass production and most importantly nutrient translocation to grains. There is a general lack of awareness in high-income countries regarding the advantages of foliar applications in terms of enhancing agricultural profitability and improving the quality of grains that can contribute to a more balanced diet for humans. Therefore, foliar application strategies and novel formulations of nutrients in agricultural systems with depleted Zn in soil should be tested. It is vital to support agrochemical-foliar research to offer farmers the best solutions while protecting economic investment.

To feed the world population with nutritious food, it is paramount to adopt new methods that effectively deliver much-needed nutrients to crop systems. In this regard, emerging nanostructures offer a means to increase the delivery of Zn to grains. Despite the increasing number of glasshouse studies, still, there is still more work to be done to validate the efficacy of application in the field. Some studies have shown that nanomaterials can be more effective than conventional fertilizers, with benefits such as the controlled release of Zn, increasing Zn-use efficiency, and reducing the loss of nutrients to the environment. Although the nature of nanomaterials is nontoxic in principle, the fast release of loaded nutrients can cause toxicity that can be difficult to reverse (Shalini et al., 2018). Therefore, more research needs to be done in the area of controlled release of micronutrients from nanostructures. This is a particular challenge since studies should be conducted *in vivo*, to replicate the internal conditions of leaf, vascular tissue, and connecting tissue responsible for delivering nutrients to grain. Physiological studies should investigate the benefits of applying Zn-foliar nanoformulations in regard to overcoming the so-called ‘push-pull’ bottleneck processes identified in the wheat plant such as leaf and nodal accumulation (Stanton et al., 2022); and Zn retranslocation and loading to edible parts of the grain - the endosperm (Kamaral et al., 2020). For this reason, it is recommended that state-of-the-art technology in microscopy and combining more sophisticated approaches such as Zn isotope tracing and synchrotron-based technologies can be used to determine the localization and movement of nanoparticles and nanomaterials in crops (Kopittke et al., 2018). A better understanding of the mechanism of Zn leaf penetration, long-distance transport and loading into the grain when supplied as nanoparticles and encapsulated in nanomaterials can shed light on the most effective agronomical method to apply foliar fertilizers in crop systems aiming to increase the nutrition of grain-derived food (**Figure 1A**).

While most studies reported on total Zn accumulation in grains, the focus of biofortification should always be on increasing the concentration of bio-available nutrients in the grain (Saha et al., 2017a; Saha et al., 2017b) This requires Zn to be transported through a series of tissues in the long-distance transport pathway to reach the edible portions of the grain. In plants, Zn mobility is tightly regulated by plasma membrane transporter-like proteins (Mu et al., 2021), hence alternative methods to facilitate diffusion through membranes can be a promising approach. Ionophores are lipophilic chelating agents that transport elements through a cell membrane in the absence of a membrane protein pore. In general, there are two types of ionophores: channel formers, which form a channel in the membrane through which ions pass; and ion carriers, which transport ions across membranes. Ionophores have been largely used to improve the feed and health of livestock. Moreover, ionophores such as quercetin present in onions, nuts, and many other vegetables, have been effective at enhancing Zn uptake *in vitro* in human intestinal Caco-2 cells (Sreenivasulu et al., 2010), which is encouraging. However, the application of foliar ionophores-like molecules for grain biofortification with nutrients has not been considered. Therefore, the investigation of whether Zn-ionophore-like molecules can facilitate the transfer of Zn to grain, and to the endosperm, through facilitated plasma membrane mechanism is a promising avenue for further research.

Looking at the future, the potential of alternative formulations containing nanostructures with multiple components that can achieve a moderate yet substantial boost on grain Zn concentrations relative to Zn alone is still in its infancy. While glasshouse studies provide valuable information on the efficacy of using alternative foliar formulations with an opportunity to understand the physiological mechanisms, testing under agronomic settings under variable agroecological conditions is imperative for validation, and most importantly for comparing such novel formulations against conventional ZnSO_4_, which is key in establishing cost margins. The adoption of a framework for comparison of agronomic methods will be beneficial for assessing the efficiency of foliar formulations against wheat varieties with variable capacity in nutrient remobilization. Therefore, a nano approach on how to use existent nanostructures and lipid-chelators for membrane transport and better compartmentalization of nutrients should help propel advances in grain Zn biofortification.

## Author contributions

RB conceived the idea of writing the paper. JTS-P accessed, revised, and collected the relevant literature and wrote the manuscript. BP and JTS-P contributed to the section on adjuvants. DH and JTS-P contributed to the sustained controlled release formulation section. JTS-P contributed to the framework model, future perspectives and conclusion sections. JTS-P, DH, BP and RB edited the final version. All authors contributed to the article and approved the submitted version.
